# Association between Blood Glucose Control and Subjective Cognitive Decline in Korean Patients with Diabetes Aged over 50 Years

**DOI:** 10.3390/ijerph19127267

**Published:** 2022-06-14

**Authors:** Dae-Hyung Koh, Yu-Jin Rho, Soon Young Lee, Kyoung-Nam Kim, Yeong Jun Ju

**Affiliations:** 1 Department of Medicine, Ajou University School of Medicine, Suwon-si 16499, Korea; eogud0716@ajou.ac.kr (D.-H.K.); ryj0910@ajou.ac.kr (Y.-J.R.); 2 Department of Preventive Medicine and Public Health, Ajou University School of Medicine, Suwon-si 16499, Korea; sole@ajou.ac.kr

**Keywords:** blood sugar control, subjective cognitive decline, Korea, diabetes, cross-sectional study

## Abstract

This study aimed to investigate the association between blood glucose control and subjective cognitive decline in adult patients with diabetes. Using the 2018 data from the community health survey, we included 18,789 patients with diabetes aged ≥50 years who had complete responses recorded. Blood glucose control was the independent variable, and subjective cognitive decline was the dependent variable. Multivariable logistic regression analysis was used to analyze the association between blood glucose control and subjective cognitive decline. Multivariable logistic regression analysis showed that blood glucose control was inversely associated with subjective cognitive decline in patients with diabetes. Patients with uncontrolled blood glucose levels had higher odds of subjective cognitive decline than those with controlled blood glucose levels (odds ratio = 1.22; 95% confidence interval: 1.10, 1.34). Our findings suggest that patients with diabetes may demonstrate subjective cognitive decline if their blood glucose levels are not well-controlled.

## 1. Introduction

With the transition to an aging society, the frequency of memory problems experienced by the aging population has been increasing [1]. Recently, 60% of patients aged ≥60 years have complained of memory problems, and subjective cognitive decline, which many older adults complain of, has been confirmed as a precursor symptom of mild cognitive impairment. Mild cognitive impairment, which is the pre-dementia stage, refers to a state in which cognitive function is inferior to that of the same age group; however, unlike dementia, the ability to perform daily life activities is preserved [2]. The identification of cognitive impairments in patients with both objective and subjective complaints, which are the hallmark features of a pre-dementia condition, is a major component in the prevention and management of dementia [3,4]. Therefore, it is important to identify factors related to subjective cognitive decline from the perspective of dementia prevention and management. 

Recently, a growing body of literature has suggested that type 2 diabetes may be considered a risk factor for mild cognitive impairment [5,6,7]; moreover, one study reported that cognitive impairment in patients with diabetes accelerated the progression to dementia [8]. Although the mechanism underlying the effect of diabetes on cognitive impairment is not clear, some studies have focused on glucose control. Unstable blood glucose levels, including higher-than-average glucose levels and increased glucose fluctuation, were reported to be significantly associated with cognitive impairment [9,10]. In addition, hyperinsulinemia due to insulin resistance was reported to increase the risk of dementia by causing the accumulation of beta-amyloid, which competitively binds to insulin to decompose enzymes and hyperphosphorylate tau proteins [11,12]. Meanwhile, the relationship between blood glucose control and cognitive impairment in patients with diabetes is controversial. Some studies have suggested that the association of blood glucose with cognitive impairment varies among study subjects [13,14], while some longitudinal studies have reported no association [15].

The recognition of the importance of blood sugar management to preventing cognitive decline in patients with diabetes is gradually increasing, but the evidence is still insufficient, and many additional studies are needed [9,16]. Hence, there is a need to further solidify the evidence based on the findings of studies conducted in various countries with sufficient sample sizes. Therefore, this study aimed to investigate the association between blood glucose control and subjective cognitive decline symptoms in Korean patients with diabetes aged ≥50 years.

## 2. Materials and Methods

### 2.1. Data and Study Population

Data from the 2018 Korea Community Health Survey (KCHS) were used in this study. The KCHS is conducted by the Korea Centers for Disease Control and Prevention. It is a cross-sectional survey, with a study population drawn from multistage, stratified area probability samples of civilian, non-institutionalized Korean households categorized according to geographic area, age, and sex. Hence, samples were representative of the wider Korean population. Additionally, people with dementia and institutionalized people were excluded because they could cause measurement uncertainty resulting from communication difficulties. Thus, all study populations comprised nondemented and noninstitutionalized Koreans. The survey is conducted annually, and data are collected through in-person (one-on-one) interviews.

**Figure 1:**
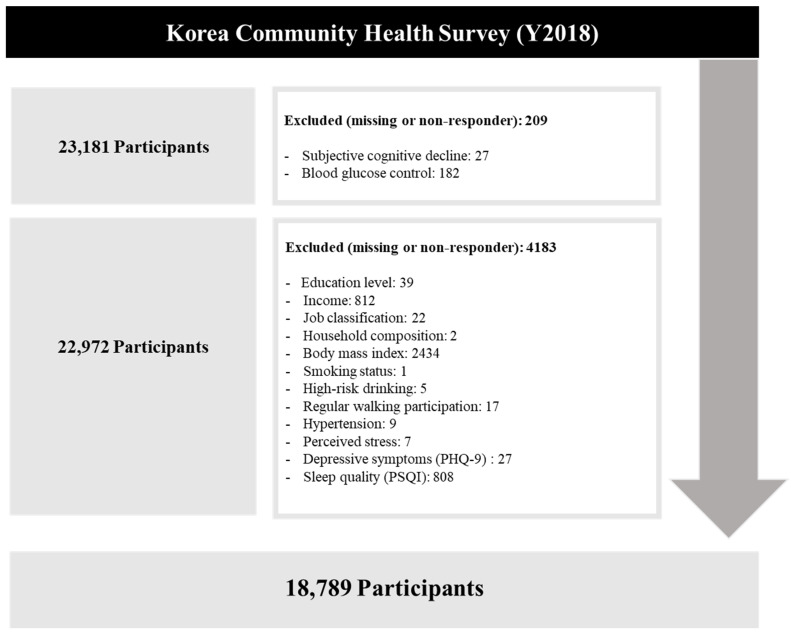
Selection of study participants.

As subjective cognitive decline was measured for participants aged ≥50 years, this study included patients with diabetes aged ≥50 years. From the initial 23,181 participants, those without data on the relevant variables were excluded. Finally, 18,789 individuals were included in the analysis (Figure 1).

### 2.2. Dependent Variable

Subjective cognitive decline was measured using the cognitive decline module of the Behavioral Risk Factor Surveillance System, which was developed by the Centers for Disease Control and Prevention [17]. Subjective cognitive decline was assessed based on the following question: “During the past 12 months, have you experienced confusion or memory loss that is happening more often or is getting worse?” Respondents who answered “yes” were considered to have subjective cognitive decline. Of note is that subjective cognitive decline was successfully evaluated based on the aforementioned question in previous investigations [18,19].

### 2.3. Independent Variable

Blood glucose control was assessed based on the following question: “Is your blood glucose well controlled?” Respondents who answered “no” were considered to have uncontrolled blood glucose levels. Of note is that blood glucose control has been successfully evaluated based on this question in a previous investigation [20].

### 2.4. Covariates

Various sociodemographic, economic, and health-related covariates were included. The variables were sex (male or female), age (50–59 years, 60–69 years, 70–79 years, or ≥80 years), educational level (none, elementary school, middle school, high school, or at least college), income (quartiles), job classification (white-collar, pink-collar, blue-collar, or unemployed), living alone (yes or no), household composition (one, two, or three generations), walking participant (yes or no), smoking status (yes or no), high-risk drinking (yes or no), body mass index (BMI; kg/m^2^), hypertension diagnosis (yes or no), depressive symptoms (yes or no), perceived stress (yes or no), sleep quality (yes or no), and subjective health status (fair or poor).

Depressive symptoms were evaluated using the Patient Health Questionnaire-9 (PHQ-9), which is commonly used to screen for depression. Sleep quality was assessed using the Pittsburgh Sleep Quality Index (PSQI). The validity and reliability of the Korean versions of the PHQ-9 and PSQI have previously been verified [21,22]. 

### 2.5. Statistical Analysis

The general characteristics of the study population were investigated using chi-square tests. Multivariable logistic regression analyses were used to determine the association between blood glucose control and subjective cognitive decline. All analyses were conducted after adjustment for the above-mentioned covariates, which were selected in accordance with related previous studies [14,16]. Additionally, the fit of the logistic model was assessed using the Hosmer–Lemeshow goodness-of-fit test, in which a well-fitting logistic model has a nonsignificant value [23]. Multicollinearity was also tested using a variance inflation factor, and we confirmed that no variables violated the multicollinearity in our model. Results are presented as odds ratios (ORs) with 95% confidence intervals (CIs). All analyses were conducted using appropriate sample weights, strata, and strata variables to consider the multistage stratified sampling design of KCHS. SPSS software (version 25.0; SPSS Inc., Chicago, IL, USA) was used for statistical analysis. The values of *p* were two-sided, and *p* < 0.05 was considered statistically significant.

### 2.6. Ethical Approval

The KCHS data are publicly available. Participant data were fully anonymized before release. Our study was excluded from the review list pursuant to Article 2.2 of the Enforcement Rule of Bioethics and Safety Act in Korea since the data were exempted from IRB review. All procedures performed in studies involving human participants were conducted in accordance with the ethical standards of the national research committee and the 1964 Helsinki Declaration and its later amendments or comparable ethical standards.

## 3. Results

The general characteristics of the study participants are shown in Table 1. This study analyzed the data of 18,789 individuals, of whom 2311 (12.3%) reported that they had experienced uncontrolled blood glucose levels. Patients with diabetes who were younger, female, less educated, with lower income, unemployed, living alone, with bad subjective health status, with higher stress, and with poor sleep quality were more likely to experience uncontrolled blood glucose levels.

Among the 18,789 participants, 13,580 (72.3%) reported no subjective cognitive decline, whereas 5209 (27.7%) reported a subjective cognitive decline. The proportion of those reporting subjective cognitive decline was higher among patients with diabetes with uncontrolled blood glucose levels (no subjective cognitive decline, 65.5%; subjective cognitive decline, 34.5%) than among diabetic patients with controlled blood glucose levels (no subjective cognitive decline, 73.2%; subjective cognitive decline, 26.8%) (Table 2).

The Hosmer–Lemeshow test was non-significant (*p* = 0.377), indicating adequate goodness-of-fit. Multivariable logistic regression analysis was used to investigate the association between blood glucose control and subjective cognitive decline, and the results are shown in Table 3. Patients with diabetes with uncontrolled blood glucose levels had a higher risk of subjective cognitive decline (odds ratio (OR) = 1.22, 95% confidence interval (CI): 1.10–1.34) than those with controlled blood glucose levels. Other than blood glucose control, the risks of subjective cognitive decline were higher among patients with diabetes who were older (OR = 1.30, 95% CI: 1.17, 1.45 for 60–69 years old; OR = 1.81, 95% CI: 1.61, 2.04 for 70–79 years old; and OR = 2.27, 95% CI: 1.96, 2.63 for ≥80 years old vs. patients aged 50–59 years), those with jobs other than white-collar jobs (OR = 1.30, 95% CI: 1.04, 1.62 for pink-collar jobs; OR = 1.36, 95% CI: 1.12, 1.66 for blue-collar jobs; and OR = 1.44 95% CI: 1.19, 1.76 for unemployed; vs. patients with white-collar jobs), with bad subjective health status (OR = 1.53, 95% CI: 1.38, 1.70; vs. patients with good subjective health status), and with poor sleep quality (OR = 1.62, 95% CI: 1.51, 1.73; vs. patients with poor sleep quality) (Table 3).

## 4. Discussion

We revealed that patients with diabetes with uncontrolled blood glucose generally have a higher likelihood of experiencing subjective cognitive decline. After adjusting for extensive potential confounders, uncontrolled blood glucose levels were associated with a higher risk of subjective cognitive decline in Korean patients with diabetes aged >50 years.

Previous studies have demonstrated that patients with diabetes have a higher risk of pathological cognitive decline and dementia than those without diabetes [24,25]. In addition, a systematic review reported some evidence on the association between blood glucose levels and the risk of dementia in individuals without diabetes, although the results demonstrated substantial heterogeneity [26]. However, to the best of our knowledge, evidence on whether uncontrolled blood glucose levels may increase the risk of increased cognitive decline in patients with diabetes is scarce. Although there is discordance between the evidence of various studies regarding the effectiveness of tight blood glucose control to reduce complications of diabetes [27], the results of this study support the value of tight blood glucose control in preventing cognitive decline. Therefore, our study addresses important knowledge gaps and has direct clinical and public health implications.

In the present study, although poor blood glucose control was associated with a higher risk of subjective cognitive decline, younger patients with diabetes were more likely to have poor glucose control, while older patients with diabetes were more likely to have subjective cognitive decline. These associations among blood glucose control, subjective cognitive decline, and age should be interpreted cautiously because we found that the risk of subjective cognitive decline was higher among patients with poor blood glucose control, regardless of age.

Several attempts to elucidate the mechanisms underlying the association between blood glucose control and subjective cognitive decline have been made. For example, the level of advanced glycation end products (AGEs), which are elevated in patients with diabetes owing to hyperglycemia, is increased in the brain tissues of patients with Alzheimer’s dementia, causing beta-amyloid aggregation or nerve fiber knot formation by promoting glycation of beta-amyloid and tau proteins [28]. Owing to the interaction between accumulated AGEs and the receptors for AGEs, beta-amyloid in the blood migrates to the brain tissue through the blood–brain barrier, thereby lowering beta-amyloid metabolism and causing Alzheimer’s disease [29,30]. Moreover, signaling between the hippocampus, which functions to store and retrieve information according to external environments, and the anterior cingulate cortex increases excessively when chronic hyperglycemia persists, resulting in decreased adaptability due to neural network hypersynchronization, which increases the risk of Alzheimer’s disease [31]. Furthermore, insulin receptors exist in the hippocampus, and increased glucose metabolism in the brain owing to insulin is involved in learning and memory, improves communication between neurons, and increases brain blood flow, thus directly protecting against dementia through beta-amyloid and tau protein cleaning [32].

This study has some limitations. First, we evaluated patient cognitive decline based on one question only, i.e., “Have you experienced increasingly frequent or severe mental confusion or memory loss in the past year?” Thus, we could not accurately identify the link between mild cognitive impairment and dementia. In addition, subjective cognitive decline was self-reported and does not imply a diagnosis of cognitive impairment. Thus, ascertaining whether the participants were cognitively impaired was not possible. Moreover, recall bias was possible because the variable was self-reported. Further studies using objective survey evaluation are required. Second, we evaluated blood glucose control based on the question, “Is your blood glucose well controlled?” Hence, it was difficult to assess the degree of control or non-control. Moreover, owing to data limitations, we could not measure the type, duration, or severity of diabetes. These possibly unobserved or unreported clinical characteristics could have influenced the cognitive impairment. Future studies should use objective measurements of blood glucose levels to address this limitation. Third, we used a cross-sectional study design; therefore, causal relationships cannot be established based on our results. For example, patients with diabetes with depressive symptoms may be more likely to neglect activities to manage blood glucose levels or complain about subjective memory decline. However, previous studies with various study designs and populations have suggested that higher blood glucose levels could exacerbate impaired cognitive function in individuals with or without diabetes [24,25,26]. Finally, this study could have yielded more meaningful findings if we had examined the association between blood glucose control and subjective cognitive decline using a conceptual framework. However, it was not possible to consider an appropriate conceptual framework because the research results are mixed according to the study participants and design. Thus, a well-designed study is needed to confirm our findings in the future.

Nevertheless, this study also has notable strengths. First, because we used the data from KCHS, which are representative of the Korean population, our results can be generalized to Korean patients with diabetes aged >50 years and possibly to adult patients with diabetes in other developed countries [33]. Second, although the number of patients with mild cognitive impairment aged >50 years has been increasing, owing to the rapidly aging population, most previous studies have investigated risk factors for increased cognitive decline in those aged 60–65 years or older. As this study included patients with diabetes in their 50s, our findings provide more meaningful information that reflects the real-world setting. Third, unlike previous studies, this study controlled for mental health outcomes, such as depressive symptoms, sleep quality, and stress perception, which are known to have a substantial influence on cognitive deficit.

## 5. Conclusions

Although patients with diabetes have been reported to have a higher risk of cognitive decline and dementia, to the best of our knowledge, no previous studies have investigated whether uncontrolled blood glucose levels may increase the risk of subjective cognitive decline in patients with diabetes. In the present study, we revealed that uncontrolled blood glucose levels were associated with a higher risk of subjective cognitive decline in Korean patients with diabetes aged > 50 years. Our findings suggest that blood glucose management should be considered while addressing the cognitive decline of patients with diabetes.

## Figures and Tables

**Table 1:** Characteristics of the study participants (*n* = 18,789).

Variables	Total		Blood Glucose Control				*p*
			Yes (*n* = 16,478, 87.7%)		No (*n* = 2311, 12.3%)		
	*n*	%	*n*	%	*n*	%	
**Age (years)**							<0.001
50–59	3873	20.6	3307	85.4	566	14.6	
60–69	6560	34.9	5742	87.5	818	12.5	
70–79	6282	33.4	5575	88.7	707	11.3	
≥80	2074	11.1	1854	89.4	220	10.6	
**Sex**							<0.001
Male	9547	50.3	8369	88.5	1088	11.5	
Female	9332	49.7	8109	86.9	1223	13.1	
**Education**							<0.001
Uneducated	3008	16.0	2608	86.7	400	13.3	
Elementary school	5615	29.9	4861	86.6	754	13.4	
Middle school	3492	18.6	3065	87.8	427	12.2	
High school	4581	24.4	4066	88.8	515	11.2	
At least college	2093	11.1	1878	89.7	215	10.3	
**Income**							<0.001
Low	7800	41.5	6717	86.1	1083	13.9	
Middle–low	5215	27.8	4596	88.1	619	11.9	
Middle–high	3293	17.5	2942	89.3	351	10.7	
High	2481	13.2	2223	89.6	258	10.4	
**Job classification**							0.002
White	1060	5.6	964	90.9	96	9.1	
Pink	1565	8.3	1379	88.1	186	11.9	
Blue	6186	32.9	5444	88.0	742	12.0	
Unemployed	9978	53.1	8691	87.1	1287	12.9	
**Living alone**							<0.001
Yes	3697	19.7	3154	85.3	543	14.7	
No	15,092	80.3	13,324	88.3	1768	11.7	
**Family composition**							0.017
One generation	13,278	70.7	11,662	87.8	1616	12.2	
Two generations	4353	23.2	3777	86.8	576	13.2	
Three generations	1158	6.1	1039	89.7	119	12.3	
**Body mass index**							0.244
<25.0	11,312	60.2	9895	87.5	1417	12.5	
≥25.0	7477	39.8	6583	88.0	894	12.0	
**Smoking**							0.014
Yes	2740	14.6	2364	86.3	376	13.7	
No	16,049	85.4	14,114	87.9	1935	12.1	
**High-risk drinking**							0.003
Yes	1840	9.8	1574	85.5	266	14.5	
No	16,949	90.2	14,904	87.9	2045	12.1	
**Participation in regular walking**							<0.001
Yes	8292	44.1	7416	89.4	876	10.6	
No	10,497	55.9	9062	86.3	1435	13.7	
**Hypertension diagnosis**							0.004
Yes	12,296	65.4	10,846	88.2	1450	11.8	
No	6493	34.6	5632	86.7	861	13.3	
**Subjective health status**							<0.001
Good	2946	15.7	2752	93.4	194	6.6	
Bad	15,843	84.3	13,726	86.6	2117	13.4	
**Perceived stress**							<0.001
Yes	4135	22.0	3242	82.8	711	17.2	
No	14,654	78.0	13,054	89.1	1600	10.9	
**Depressive symptoms (PHQ-9 score)**							<0.001
Yes (≥10)	1102	5.9	815	74.0	287	26.0	
No (<10)	17,687	94.1	15,663	88.6	2024	11.4	
**Sleep quality (PSQI score)**							<0.001
Good (≤5)	9682	51.5	8790	90.8	892	9.2	
Poor (>5)	9107	48.5	7688	84.4	1419	15.6	

PHQ-9, Patient Health Questionnaire-9; PSQI, Pittsburgh Sleep Quality Index.

**Table 2:** Study participants stratified by subjective cognitive decline and blood glucose control (*N* = 18,789).

	Total		Subjective Cognitive Decline				*p*
			No (*n* = 13,580, 72.3%)		Yes (*n* = 5209, 27.7%)		
	*n*	%	*n*	%	*n*	%	
**Blood glucose control**							<0.001
Controlled group	16,478	87.7	12,066	73.2	4412	26.8	
Uncontrolled group	2311	12.3	1514	65.5	797	34.5	

**Table 3:** Results of multivariable logistic regression analysis ^1^ of the association between blood glucose control and subjective cognitive decline.

Variables	Subjective Cognitive Decline		
	Adjusted OR ^1^	95% CI	
**Blood glucose control**			
Controlled group	1.00.		
Uncontrolled group	1.22	1.10	1.34
**Age (years)**			
50–59	1.00		
60–69	1.30	1.17	1.45
70–79	1.81	1.61	2.04
≥80	2.27	1.96	2.63
**Sex**			
Male	1.00		
Female	1.06	0.97	1.15
**Education**			
Uneducated	1.00		
Elementary school	1.11	0.92	1.13
Middle school	0.97	0.86	1.09
High school	0.89	0.78	1.00
At least college	0.98	0.84	1.14
**Income**			
Low	1.00		
Middle–low	1.04	0.95	1.13
Middle–high	0.98	0.88	1.10
High	0.97	0.84	1.11
**Job classification**			
White	1.00		
Pink	1.30	1.04	1.62
Blue	1.36	1.12	1.66
Unemployed	1.44	1.19	1.76
**Living alone**			
Yes	1.00		
No	0.96	0.88	1.06
**Family composition**			
One generation	1.00		
Two generations	1.05	0.96	1.15
Three generations	1.19	1.02	1.38
**Body mass index**			
<25.0	1.00		
≥25.0	0.96	0.90	1.03
**Smoking**			
Yes	1.00		
No	1.09	0.98	1.22
**High-risk drinking**			
Yes	1.00		
No	0.95	0.83	1.08
**Participation in regular walking**			
Yes	1.00		
No	1.03	0.96	1.10
**Hypertension diagnosis**			
Yes	1.00		
No	1.03	0.95	1.10
**Subjective health status**			
Good	1.00		
Bad	1.53	1.38	1.70
**Perceived stress**			
Yes	1.00		
No	0.75	0.70	0.82
**Depressive symptoms (PHQ-9 score)**			
Yes (≥10)	1.00		
No (<10)	0.38	0.33	0.44
**Sleep quality (PSQI score)**			
Good (≤5)	1.00		
Poor (>5)	1.62	1.51	1.73

OR, odds ratio; CI, confidence interval; PHQ-9, Patient Health Questionnaire-9; PSQI, Pittsburgh Sleep Quality Index. ^1^ Adjusted for age, sex, education, income, job classification, living alone, family composition, body mass index, smoking, high-risk drinking, participation in regular walking, hypertension diagnosis, subjective health status, perceived stress, depressive symptoms, and sleep quality.

## Data Availability

All the Korea Community Health Survey data used in this study are available to the public and can be accessed on the Korea Community Health Survey website (https://chs.kdca.go.kr/chs/rdr/rdrInfoProcessMain.do, accessed on 1 June 2022).
